# Meta-Analysis of Vaginal Microbiome Data Provides New Insights Into Preterm Birth

**DOI:** 10.3389/fmicb.2020.00476

**Published:** 2020-04-08

**Authors:** Idit Kosti, Svetlana Lyalina, Katherine S. Pollard, Atul J. Butte, Marina Sirota

**Affiliations:** ^1^Bakar Computational Health Sciences Institute, University of California, San Francisco, San Francisco, CA, United States; ^2^Department of Pediatrics, University of California, San Francisco, San Francisco, CA, United States; ^3^Integrative Program in Quantitative Biology, Gladstone Institutes, University of California, San Francisco, San Francisco, CA, United States; ^4^Department of Epidemiology & Biostatistics, Institute for Human Genetics, University of California, San Francisco, San Francisco, CA, United States; ^5^Quantitative Biosciences Institute, University of California, San Francisco, San Francisco, CA, United States; ^5^Gladstone Institutes, San Francisco, CA, United States; ^6^Chan Zuckerberg Biohub, San Francisco, CA, United States

**Keywords:** microbiome, vaginal microbiome, preterm birth, longitudinal analysis, meta-analysis, public data analysis

## Abstract

Preterm birth (PTB) is defined as the birth of an infant before 37 weeks of gestational age. It is the leading cause of perinatal morbidity and mortality worldwide. In this study, we present a comprehensive meta-analysis of vaginal microbiome in PTB. We integrated raw longitudinal 16S rRNA vaginal microbiome data from five independent studies across 3,201 samples and were able to gain new insights into the vaginal microbiome state in women who deliver preterm in comparison to those who deliver at term. We found that women who deliver prematurely show higher within-sample variance in vaginal microbiome abundance, with the most significant difference observed during the first trimester. Modeling the data longitudinally revealed a number of microbial genera as associated with PTB, including several that were previously known and two newly identified by this meta-analysis: *Olsenella* and *Clostridium sensu stricto*. New hypotheses emerging from this integrative analysis can lead to novel diagnostics to identify women who are at higher risk for PTB and potentially inform new therapeutic interventions.

## Introduction

Preterm birth (PTB) is defined as a live birth before 37 weeks of gestational age. According to the Centers of Disease Control and prevention, one of every 10 infants born in the United States is born prematurely (Ferré, 2016). PTB and low birth weight are among the top causes for infant death in the United States (Mathews and MacDorman, 2010), and can cause complications to newborns (Ward and Beachy, 2003). Despite many attempts for PTB prevention, there is still an acute problem with prevalence rising according to the World Health Organization (Beck et al., 2010).

Spontaneous PTB, accounting for two-thirds of all PTBs (Goldenberg et al., 2008; Romero et al., 2014a), is considered a complex phenotype which can arise due to different causes. Risk factors for spontaneous preterm birth include a previous preterm birth, race, periodontal disease, low maternal body-mass index (Goldenberg et al., 2008), maternal stress (Copper et al., 1996), together with other demographic characteristics such as maternal age, low socioeconomic and educational status and marital status (Goldenberg et al., 2008). In a recent analysis carried out on a large cohort, maternal age of over 40 was associated with PTB (Fuchs et al., 2018). The mechanism behind PTB is unknown, but the phenomenon appears to be a collection of similar phenotypes with many different mechanisms. One proven mechanism is chorioamnionitis, a condition associated with microbial infection of the amniotic fluids (Romero et al., 2001). Unfortunately, intermittent antibiotic treatment of non-pregnant women who had an early spontaneous PTB did not significantly reduce subsequent PTBs (Andrews et al., 2006). Other suggested causes of PTB are progesterone deficiency, disruption of the immune tolerance of the mother toward the fetus and disruption of the vaginal microbial balance, causing an inflammatory process (Romero et al., 2014a), which is the focus of our study.

The human microbiome is becoming a major area of interest with several large consortiums such as the Human Microbiome Project releasing large reference datasets (Turnbaugh et al., 2007). The main focus currently is gut microbiota, but microbial communities from other body sites (oral, vaginal, and skin) are also being studied (Lloyd-Price et al., 2016). In general, the vaginal microbiome is less diverse in comparison to other body sites (Hyman et al., 2005), especially during pregnancy (Aagaard et al., 2012; Romero et al., 2014a). One main focus area of vaginal microbiome research is Bacterial vaginosis (BV), which is a risk factor for PTB (Nejad and Shafaie, 2008). A *Lactobacilli*-dominated microbiome is associated with a healthy state and BV is best described as a poly-bacterial instability state, with a shift toward higher concentrations of anaerobic bacteria. This high microbial diversity is associated with pro-inflammatory genital cytokines (Anahtar et al., 2015). Romero et al. (2014c) showed in a longitudinal study that pregnant women have a higher abundance of *Lactobacilli* compared to non-pregnant women. Others reported that the vaginal microbiome varies by gestational age and proximity to the cervix (Aagaard et al., 2012).

Studying the differences in vaginal microbiome during pregnancy with respect to birth timing may provide better understanding of the role of vaginal microbiome in PTB. Romero et al. (2014b) did not find any significant vaginal microbiome differences longitudinally. Hyman et al. (2014) found that uncultured bacteria are significantly different during term and PTB pregnancies, and that black patients have the most diverse microbial communities. DiGiulio et al. (2015) found several differences in abundance in the vaginal microbiome between women who deliver prematurely in comparison to those who deliver at term. They show that women with a *Lactobacilli*-poor community together with elevated *Gardnerella* or *Ureaplasma* abundances had a higher chance to deliver prematurely. Callahan et al. (2017) were able to confirm these associations in one of the cohorts that they examined. Stout et al. (2017) found that in a predominantly black population, there is higher vaginal microbial community richness and diversity in the first and second trimester of pregnancy in women who delivered prematurely when compared to women who delivered at term. Since the previous studies do not have consistent results and vary in the number of samples, subjects and ethnic backgrounds, we aimed to perform a meta-analysis to address these issues and see whether there is a common microbial signature associated with PTB.

Meta-analysis is a systematic approach to combine and integrate cohorts to study a biological question or a disease condition (Haidich, 2010). Meta-analysis provides enhanced statistical power due to a higher number of samples when combined. On the other hand, it requires care to ensure that data are comparable across cohorts and hence commonly utilizes methods such as batch correction (Chen et al., 2011) and mixed effect modeling (Stram, 1996). Meta-analysis has been carried out extensively in the fields of gene expression (Waldron and Riester, 2016) and genome-wide association studies (Evangelou and Ioannidis, 2013), where the robustness of the signatures across different studies is well established (Chen et al., 2014; Hughey and Butte, 2015). Meta-analysis across microbiome studies is much less common due to heterogeneity of the data and lack of analytical standards, though a few recent publications show that meta-analysis is possible and advantageous for microbiome data (Koren et al., 2013; Henschel et al., 2015; Duvallet et al., 2017; Mancabelli et al., 2017). In a recent paper by Duvallet et al. (2017), the authors demonstrate the importance of performing meta-analysis for gut microbiome in health and disease across a large number of studies and samples. Haque et al. (2017) present summary-level meta-analysis using pre-calculated abundance tables from the original studies to draw conclusions about the combined studies. They found that there is a difference in the variance of the vaginal microbiome in the first trimester between women who delivered prematurely and at term. In this study, we perform a comprehensive meta-analysis across 3,201 vaginal microbiome samples from 415 patients. We re-analyze all the samples with the same pipeline, correct for batch effects and apply a longitudinal modeling approach to demonstrate that publicly available data and robust computational approaches can be leveraged to identity new associations between bacterial genera and PTB. A web application with all the analysis results of this study is also available here: https://comphealth.ucsf.edu/app/ptb_microbiome_metaanalysis.

## Materials and Methods

### Data Availability

Raw data and metadata for the DiGiulio et al. (2015) cohort were downloaded from ImmPort (Bhattacharya et al., 2014), under Study SDY465 (Immport, 2011) in May 2016. Raw data and metadata for Romero et al. (2014b) cohort were downloaded from the Sequence Read Archive (Leinonen et al., 2011) under BioProject PRJNA242473 (NCBI Sequence Read Archive, 2014)^1^ in May 2016. Raw data for Hyman et al. (2014) cohort were received from the authors of the study (raw sequences and weeks of collection), metadata was downloaded from Supplementary Figure S1 of the paper. Raw data and metadata for the Callahan et al. (2017) cohort were downloaded from the Sequence Read Archive under BioProject PRJNA393472 (NCBI Sequence Read Archive, 2014)^2^ in January 2018. Raw data and metadata for the Stout et al. (2017) cohort were downloaded from the Sequence Read Archive under BioProject PRJNA294119 (NCBI Sequence Read Archive, 2014)^3^ in January 2018. The processed data was uploaded to ImmPort (Bhattacharya et al., 2014) under study SDY1162 (Immport, 2019) and to figshare (Kosti et al., 2019), together with relevant metadata. The DiGiulio cohort were collected from various locations within the United States. DiGiulio cohort was collected at Stanford University (California), the Callahan cohort was collected at Stanford University (California) and UAB (Alabama), the Romero cohort was collected at Wayne State University (Michigan), the Hyman cohort was collected at UCSF (California) and the Stout cohort was collected at Washington University in St. Louis (Missouri).

### Definition of Trimesters in Study Cohorts

The raw sequences from the five studies mentioned above were filtered based on their metadata body site information, keeping only sequences from vaginal samples. For each cohort, the trimester of collection was defined by the following guidelines: samples taken in weeks 9–13 are defined as first trimester samples, samples taken in weeks 14–25 are defined as second trimester samples and weeks 26–36 are defined as third trimester samples. We exclude all samples before week 9 and after week 36 from our analysis, to assure similar distributions of sampling in PTB and term cohorts.

### UPARSE Pipeline

**FIGURE 1:**
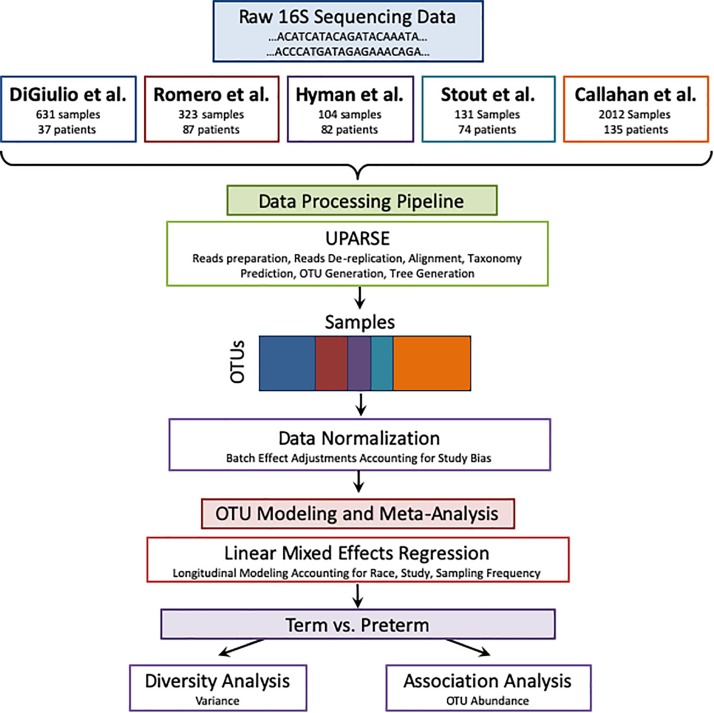
Meta-analysis pipeline. The pipeline is divided into four sections: combining raw 16S rRNA data from five publicly available studies, processing the data using UPARSE, data normalization, and modeling using LMER to analyze with respect to birth timing as an outcome.

The pipeline receives as input raw 16S sequences collected from different studies as shown in Figure 1. It also receives the metadata from all cohorts according to shared properties: Sample and patient ID, week of specimen collection, body site of collected specimen, delivery outcome (term or PTB), trimester of pregnancy (as mentioned above), and patient’s race. The sequences are entered into a UPARSE OTU analysis pipeline using the USEARCH algorithm (Edgar, 2013). The following steps are carried out by USEARCH: (1) reads preparation, (2) reads de-replication by recognizing unique sequences, removing non-biological sequences and removing singletons, (3) taxonomy prediction using 16S reference set from RDP (Wang et al., 2007) (4) Generating an OTU table (5) tree creation by agglomerative clustering of reads. The reason to choose an OTU based method (over denoising methods) is the data in use in this meta-analysis. Earlier data, such as the Hyman cohort could only be found in FASTA format. To address the amplification by different primers, we carried out closed reference alignment of the data to ensure the robustness of the results and concordance of the studies. The closed-reference OTU picking process is the approach of choice if non-overlapping amplicons are compared.

### Data Normalization

**FIGURE 2:**
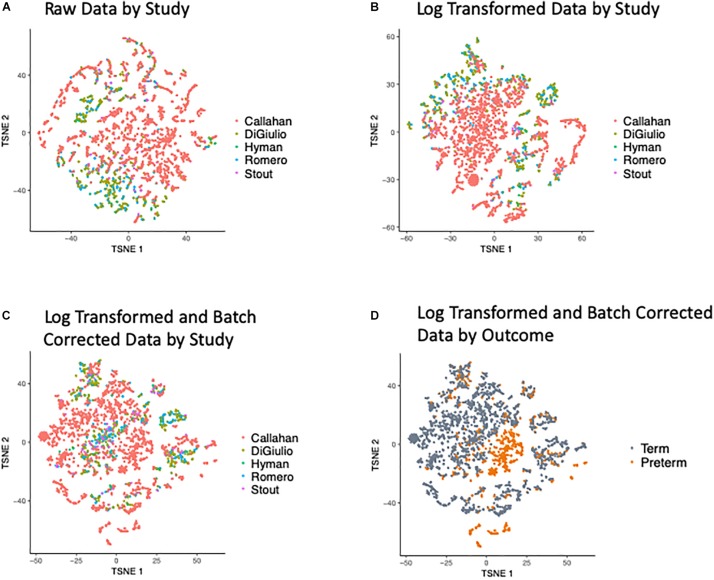
t-SNE Plots of Term and Preterm OTUs. Coloring of the t-SNE Plots is by: (1) Data Origin for **(A)** raw data, **(B)** data after log transformation, **(C)** data after log transformation and ComBat modeling. (2) Pregnancy Outcome **(D)** data after log transformation and ComBat modeling.

Our first effort was directed at processing the five input data sets to ensure these were compatible for cross-dataset analysis and integration. t-Distributed Stochastic Neighbor Embedding (t-SNE) of the OTUs revealed a bias in the data with samples clustering within the original studies (as shown in Figure 2A), when we look at the abundance of the OTUs (counts per million) and after applying a Log transformation (Figure 2B). To overcome this bias we applied the empirical Bayes algorithm ComBat (Johnson et al., 2007) from the sva R package (Leek et al., 2012) that was originally designed to remove batch effects from gene expression microarray measurements. The results of the parametric empirical Bayes data adjustment on the log transformed OTUs is shown in Figure 2C. We used gPCA R package, based on Reese et al. (2013), to show batch removal from the data after passing ComBat correction, using the test statistic delta (which describes batch effect magnitude).

### Downstream Filtering of the OTU Table Before Applying Weighted Model on OTUs

The OTU table was filtered to exclude the bottom 30% of least abundant OTUs, resulting in 8,538 OTUs. This step was taken because including low abundance OTUs in the parallel model fitting procedure led to convergence failures. The threshold was chosen empirically using an analysis of progressively higher thresholds and stopping when the convergence problem was fixed. While the exclusion of OTUs below this quantile may lead to us missing promising rare bacteria, this data restriction does not increase type I error and was performed primarily for technical convenience rather than to enrich for discoveries. Omitting consistently low count OTU may also help remove OTUs that arise from biases in the input data or OTU identification procedure.

### Data Aggregation by Taxonomy

The raw OTU table was aggregated by the taxonomic assignment of UPARSE. For each genus, we sum all OTUs assigned to it, resulting in 690 genera. We than applied log transformation followed by ComBat on the data as mentioned above.

### Within-Sample Variance Calculation, Modeling and Statistics

In order to show correlation of evenness with within-sample variance, both parameters were calculated on the non-normalized data, within each dataset.

The var function in *R* was used to calculate the within-sample variance in the following manner: the sum of squared deviations of every observation from the sample mean, divided by the degrees of freedom.

(1) $$s^{2}=\frac{\sum_{i=1}^{n}x_{i}-\bar{X}}{n-1}$$

variance equation

Where *s*^2^is the variance, *n* is the number of samples, *x*_*i*_ is the value per sample and $\bar{X}$ is the sample mean.

Evenness (*J*) is defined as the diversity based on Simpson method (*H*) over total number of non-zero OTUs in a sample (S).

(2) $$J=\frac{H}{l\,n\,\left(S\right)}$$

Evenness equation

The diversity was calculated using the vegan R package.

Within-sample variance of OTU abundance was calculated for overall term and preterm samples, and for term and preterm samples in the first, second and third trimesters separately using *R*’s var function. The within-sample variance calculation shows how variable are the normalized counts from the mean for that particular sample. The calculation was performed for each sample individually, on the ComBat normalized data.

For data from term and preterm pregnancies plotted by weeks a local polynomial regression was fitted, and the Kolmogorov–Smirnov statistic was used to test for significant differences between distributions. The data was also modeled to correct for difference between sampling strategies cohorts and racial composition using linear mixed effects regression from lme4 package in R (Bates et al., 2015).

For within-sample variance analysis by outcome we used the following model:

Variance∼Trimester+Outcome+Trimester:Outcome+Race+(1|subjectID)

Trimester represents the sample’s trimester of collection, Outcome represents the sample’s phenotype (term or preterm), Race represents the race of the patient (white, black, asian, or other) and subjectID represents the subject ID for each sample. The p-values were obtained by Satterthwaite approximation from the model coefficients using the lmerTest R package (Kuznetsova et al., 2017).

### Data Modeling for OTUs

Weighted linear mixed effects regression with the lme4 (v1.1-15) R package was used to test for differences in OTU abundance between term and preterm vaginal swab samples. For each of OTU the following model was fit:

Log⁢(read⁢counts⁢per⁢million)∼Trimester+Outcome+Trimester:Outcome+Race+(1|subjectID)+(1|Study)

Trimester represents the sample’s trimester of collection, Outcome represents the sample’s phenotype (term or preterm), Race represents the race of the patient (white, black, asian, or other), subjectID represents the subject ID for each sample and Source is the sample’s original cohort (DiGiulio, Romero, Callahan, Stout, or Hyman).

The weights used were obtained by running the voom function in limma (v3.30.13) (Ritchie et al., 2015) with a similar regression formula, minus the random effects. Contrasts of interest were tested with the emmeans package (v1.1), which allowed us to find group differences conditional on the trimester of observation and averaging over the effect of race. The log fold change we report represents the average change in logCPM when comparing term and preterm samples: log(CPM_preterm/CPM_term). The *p*-values from all trimesters and OTUs were then adjusted for multiple testing using the Benjamini-Hochberg procedure as implemented in the “p.adjust” function in R.

### Code Availability

R custom code used to generate the figures and analysis in the meta-analysis can be found on figshare (Kosti et al., 2019). An Rdata file with computed summary counts tables and other necessary input files for the code (such as metadata) is also available on figshare (Kosti et al., 2019).

## Results

### Study Cohorts

We searched the literature for raw 16S data from publicly available microbiome studies from vaginal samples of pregnant women who went on to deliver either preterm or at term. We excluded studies with only processed data or studies lacking metadata and outcome information. Five studies met our criteria: Hyman et al. (2014), Romero et al. (2014b), DiGiulio et al. (2015), Callahan et al. (2017), and Stout et al. (2017)(Table 1). To our knowledge there were no other publicly available PTB related microbiome studies that meet the above criteria at the time of study design. The prevalence of PTB in all five cohorts (ranging from 12.5 to 37%) is higher than in the general population, likely reflecting study design and clinical settings (Ferré, 2016). The experimental design and sampling strategy was different for each cohort (as listed in Table 1) yielding a different number of overall samples. For all three cohorts, the most samples were collected in the second and third trimesters (defined as 14–25 and 25–37 weeks of gestation, respectively). The five cohorts are different from one another not only in their sampling study design and number of patients but also in the racial composition of the cohorts (Table 1 and Supplementary Figure S1). In the Hyman et al. (2014) and DiGiulio et al. (2015) cohorts, most samples are from white patients, while in the Romero et al. (2014b), Callahan et al. (2017), and Stout et al. (2017) cohorts, most samples are from black patients (Supplementary Figure S1). By combining these groups together in a meta-analysis (see section “Materials and Methods”), we are able to capture variability across a more diverse population of patients. The combined data set contains vaginal 16S sequences and metadata for 3,201 samples from 415 pregnant women, mostly split between black and white individuals (44 and 34% accordingly).

**TABLE 1:** General properties of individual studies included in the meta-analysis: vaginal microbiome of five cohorts of term and preterm patients: Hyman et al. (2014), Romero et al. (2014b), DiGiulio et al. (2015), Callahan et al. (2017), and Stout et al. (2017).

	Callahan et al., 2017	DiGiulio et al., 2015	Hyman et al., 2014	Romero et al., 2014b	Stout et al., 2017
Number of participants	135	37	82	87	74
Number sampled while pregnant	135	37	82	87	74
Number delivering preterm	50	5	16	18	23
Overall preterm ratio	37%	12.5%	21%	17.33%	31.1%
Number sampled during trimester 1	42 (10 PTB)	21 (4 PTB)	37 (5 PTB)	6 (2 PTB)	14 (4 PTB)
Number sampled during trimester 2	135 (50 PTB)	31 (4 PTB)	50 (10 PTB)	76 (17 PTB)	55 (18 PTB)
Number sampled during trimester 3	123 (39 PTB)	36 (4 PTB)	46 (9 PTB)	60 (5 PTB)	59 (17 PTB)
Sampling time points	One per week	One per week	One per trimester	One every 4 weeks (<24 GW) One every 2 weeks (>24 GW)	One per trimester
Number of samples collected during trimester 1	116 (28 PTB)	58 (12 PTB)	27 (4 PTB)	4 (2 PTB)	15 (4 PTB)
Number of samples collected during trimester 2	987 (310 PTB)	303 (46 PTB)	49 (10 PTB)	178 (47 PTB)	56 (19 PTB)
Number of samples collected during trimester 3	909 (204 PTB)	270 (21 PTB)	28 (8 PTB)	141 (7 PTB)	60 (18 PTB)
Number of samples from white individuals	578 (65 PTB)	413 (45 PTB)	51 (11 PTB)	14 (4 PTB)	33 (9 PTB)
Number of samples from black individuals	1004 (356 PTB)	16 (0 PTB)	8 (2 PTB)	281 (52 PTB)	88 (30 PTB)
Number of samples from Asian individuals	110 (15 PTB)	45 (0 PTB)	17 (5 PTB)	5 (0 PTB)	0 (0 PTB)
Number of samples from other race individuals	267 (95 PTB)	157 (34 PTB)	28 (4 PTB)	28 (4 PTB)	10 (2 PTB)

### Analytical Pipeline Overview

We quantified the taxonomic composition of all vaginal microbiome samples with a consistent 16S pipeline that estimates the relative abundance of each species-level operational taxonomic unit (OTU) in each sample (Figure 1; see section “Materials and Methods”). As expected, samples cluster by cohort if no normalization is applied to the OTU abundances (Figure 2A) or if log transformation is applied (Figure 2B), but this bias is gone after batch normalization (Figure 2C; see section “Materials and Methods”). We also used a guided PCA analysis (see section “Materials and Methods”) after batch removal by ComBat, resulting in the test statistic delta of 0.05 (when 1 represents full batch effect) and *p*-value < 0.01. This result means no batch effect was indicated after ComBat. To confirm the successful removal of cohort bias, we fit a linear mixed effect model to the corrected data and did not observe any significant associations between individual species and a specific cohort. We observe a partial clustering of samples by outcome based on their overall OTU abundance profiles (Figure 2D), however, there was no obvious clustering by race or trimester of collection (Supplementary Figures S2A,B). We then aggregated the data by genera and taxonomic assignment to each OTU (see section “Materials and Methods”), and repeated the same normalization process to ensure that there is no bias toward original studies and observed comparable results (Supplementary Figure S3).

### Higher Microbial Within-Sample Variance Is Observed in Women Who Delivered Prematurely and Is Consistent Across Racial Groups

Recent attempts to compare microbiome diversity between women who deliver at term and preterm has resulted in mixed findings with some studies reporting a higher diversity in women who delivered preterm (Haque et al., 2017; Stout et al., 2017) and others showing no change in diversity (Romero et al., 2014b). We looked at the microbial variability across samples with different pregnancy outcomes. To explore this, we computed the within-sample variance of OTU abundances for each sample, as an extension of previous uses of variance. In general, variance is a commonly employed metric in gene expression analysis (Pritchard et al., 2001; Mar et al., 2011) and usually is in use to compare genes across samples. It has also been used in microbiome analysis to compare individuals with different clinical outcome (Reddy et al., 2018). The within-sample variance represents how much the normalized OTU abundances for that sample differ from the sample’s mean value (see section “Materials and Methods”). We chose to use within-sample variance to describe OTU abundance for several reasons. First, we seek to quantify how uniform abundances are across OTUs for a given sample, because evenness and other metrics of alpha diversity have associated with microbiome health (Turnbaugh et al., 2009). Supplementary Figure S4 shows the correlation between evenness and variance within each of the dataset, before batch removal. In all datasets we see a negative significant correlation between the two parameters, as expected. Second, the data transformation that we used to correct for batch effects produces an OTU matrix whose entries are no longer read counts. Therefore, richness and the Shannon index–commonly employed measures of alpha diversity–are not appropriate to use here.

**FIGURE 3:**
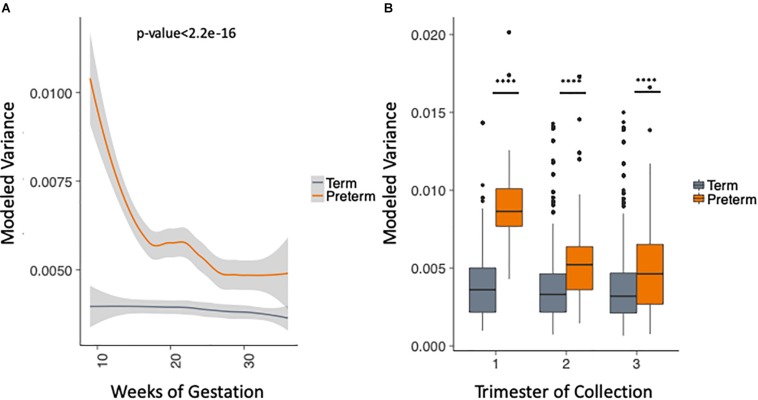
Analysis of modeled within-sample variance in PTB and term samples. **(A)** Modeled within-sample variance by weeks of gestation (trimesters 1–3). **(B)** Modeled within-sample variance by trimester of collection. The modeling takes into account the race, outcome, trimester of collection and the interaction between outcome and trimester of collection, while correcting for number of samples per patient.

We took advantage of the rich longitudinal cohort that we obtained and compared the within-sample variance across the three trimesters in women who delivered at term and preterm using a mixed linear effects regression (see section “Materials and Methods”). In this model, we take into account the trimester of collection, race and the delivery outcome, while correcting for the study and sampling frequency (see section “Materials and Methods”). The longitudinal trend of microbiome within-sample variance differs between samples from women who deliver at term and preterm (Figure 3A), with higher OTU variability across trimesters in the PTB group when compared to the term group throughout pregnancy [trimesters 1–3 (weeks 9–36); *p*-value < 2.2e-16, Two-sample Kolmogorov–Smirnov test] with the biggest difference in the first trimester (Figure 3B). We repeated the same analysis for the data aggregated by genera (see section “Materials and Methods”) and observed consistent results (Supplementary Figure S5). This finding also appears in four out of the five cohorts individually (Supplementary Figure S6, DiGiulio *p*-value < 2.2e-16, Romero *p*-value = 2.8e-9, Callahan *p*-value < 2.2e-16, and Stout *p*-value = 0.01, two-sample Kolmogorov–Smirnov test). The difference is not observed in Hyman et al. (2014) where we see very low within-sample variance in both sets, with patients who deliver at term having slightly higher within-sample variance (*p*-value = 1.1e-4, two-sample Kolmogorov–Smirnov test). It is important to note that the overall within-sample variance for this cohort is very low compared to the other cohorts and the magnitude of the difference is very small. Those results show that genus level trends are in agreement with species level trends although the V region targeted is different for the five original cohorts.

**FIGURE 4:**
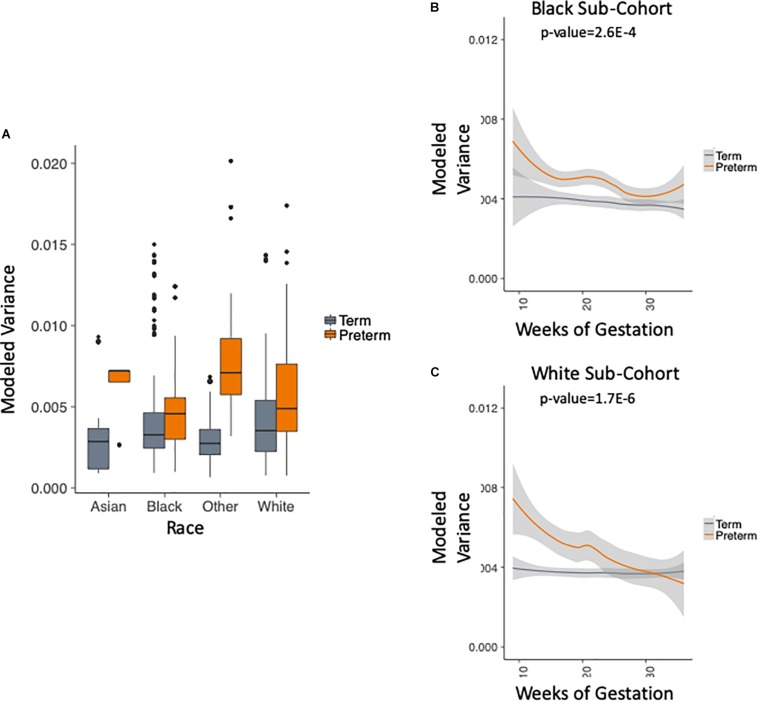
Analysis of modeled variance in different racial groups. **(A)** Modeled within-sample variance stratified by race (trimesters 1–3). **(B)** Modeled within-sample variance by weeks of gestation (trimesters 1–3) and outcome in black patients. **(C)** Modeled within-sample variance by weeks of gestation (trimesters 1–3) and outcome in white patients.

We use the metadata of the five studies to subset the data into four racial groups: white, black, asian and other. Figure 4A shows that the trend of higher within-sample variance in the PTB group is true consistently across different racial groups. We chose to exclude two groups in this analysis: (1) the asian group that had no PTB samples in the first trimester (as can be seen in Table 1); (2) the “other” group, as the race composition in the group is unknown. We then plotted the results longitudinally. Our method shows that the higher within-sample variance trend in PTB is found across different racial groups. The results are shown in Figure 4B for black patients (*p*-value = 2.6E-4, two-sample Kolmogorov–Smirnov test) and Figure 4C for white patients (*p*-value = 1.7E-6, two-sample Kolmogorov–Smirnov test).

### Vaginal Microbiome Meta-Analysis Uncovers Novel Bacterial Genera Associated With PTB

Finally, we performed an association meta-analysis comparing the abundance of the bacterial species in women who deliver preterm vs. at term. It is important to recognize that in our final cohort there is a bias arising from the difference in sampling time and study design between combined studies. The DiGiulio et al. (2015) and Callahan et al. (2017) cohorts are the largest cohorts, with the samples collected weekly from the patients whereas the Hyman et al. (2014) and Stout et al. (2017) are smaller, with one sample collected per trimester. In order to address the potential bias arising as a result of combining cohorts with different design and sampling strategy, we assessed the differences between term and preterm samples for each OTU by applying a weighted linear mixed effects regression model correcting for study, sampling bias, race (see section “Materials and Methods”) and adjusting the *p*-values by FDR.

Tables 2–4 show genera labels for OTUs that we identify as significantly associated with PTB in the first, second, and third trimesters, respectively. The abundance of these OTUs are shown in Supplementary Figure S7. Across all trimesters, there was only one OTU (Otu4172), *Lactobacillus*, that was more prevalent in patients who deliver at term. Other than this specific OTU, all the significant OTUs were more abundant in patients who delivered prematurely. In the first trimester, we were able to identify the association of a total number of six bacterial genera with PTB, shown in Table 2: *Olsenella, Dialister, Prevotella, Megasphaera, Lactobacillus*, and *Atopobium*. Five of the bacterial genera were reported previously in all or some of the original cohorts as having a higher abundance in those who deliver preterm. We also found one novel association in the first trimester using our method: *Olsenella. Olsenella* is a known oral bacterial genus (Dewhirst et al., 2010) and reported in the past as associated with BV (Srinivasan and Fredricks, 2008) but not PTB. In the second trimester, we were able to show the association of one bacterial genus with PTB, shown in Table 3: *Lactobacillus*. In the third trimester, we were able to show the association of three bacterial genera with PTB, shown in Table 3: *Gardnerella, Lactobacillus*, and *Aerococcus*. Our method now confirmed those genera as significantly associated with PTB. We also found one completely novel association using our method: *Clostridium sensu stricto* in the third trimester. There are reports of other strains of the *Clostridiales* order as involved in BV and PTB (Fredricks et al., 2005), but there is no report in the literature of the association of *Clostridium sensu stricto* with either phenotype.

**TABLE 2:** Significant bacterial OTUs in first trimester in PTB vs. term samples.

OTU	Genera	Log fold change	Adj. *P*-value	DiGiulio	Romero	Hyman	Callahan	Stout
Otu18735	*Atopobium*	0.143	7.99E-26				Significant	Reported
Otu4548	*Lactobacillus*	0.295	2.84E-23	Reported	Reported	Reported		
Otu7770	*Prevotella*	0.14	4.11E-17	Reported	Reported	Reported	Reported	Reported
Otu4172	*Lactobacillus*	−8.00E-09	9.06E-16	Reported	Reported	Reported		
Otu875	*Olsenella*	0.38	4.39E-09					
Otu5055	*Prevotella*	0.155	1.00E-06	Reported	Reported		Reported	Reported
Otu10435	*Prevotella*	0.11	1.90E-05	Reported	Reported		Reported	Reported
Otu6000	*Prevotella*	0.135	1.45E-04	Reported	Reported	Reported	Reported	Reported
Otu507	*Megasphaera*	0.75	1.92E-02	Reported	Reported	Reported	Reported	
Otu630	*Dialister*	0.46	1.92E-02				Significant	

*For each OTU we show the internal OTU label, the genera label, log fold change between preterm and term models (where positive values show a higher change in preterm), the adjusted p-value by FDR (see section “Materials and Methods”) for the model and whether the taxa were “Significant” or “Reported” in the original cohorts. “Significant” represents a reported significant difference between term and PTB abundance. “Reported” represents abundance measurement reported in the original cohorts in the original papers.*

**TABLE 3:** Significant bacterial OTUs in second trimester in PTB vs. term samples.

OTU	Genera	Log fold change	Adj. *P*-value	DiGiulio	Romero	Hyman	Callahan	Stout
Otu4172	*Lactobacillus*	−8.00E-09	9.06E-16	Reported	Reported	Reported		
Otu5546	*Lactobacillus*	0.185	2.96E-02	Reported	Reported	Reported		

*For each OTU we show the internal OTU label, the genera label, log fold change between preterm and term models (where positive values show a higher change in preterm), the adjusted p-value by FDR (see section “Materials and Methods”) for the model and whether the taxa were “Significant” or “Reported” in the original cohorts. “Significant” represents a reported significant difference between term and PTB abundance. “Reported” represents abundance measurement reported in the original cohorts in the original papers.*

**TABLE 4:** Significant bacterial OTUs in third trimester in PTB vs. term samples.

OTU	Genera	Log fold change	Adj. *P*-value	DiGiulio	Romero	Hyman	Callahan	Stout
Otu4172	*Lactobacillus*	−8.00E-09	9.06E-16	Reported	Reported	Reported		
Otu868	*Gardnerella*	0.11	3.24E-04	Significant	Reported		Significant	
Otu1238	*Clostridium sensu stricto*	0.03	1.58E-03					
Otu10429	*Aerococcus*	0.02	1.64E-02	Reported				
Otu22110	*Lactobacillus*	0.11	2.50E-02	Reported	Reported	Reported		

*For each OTU we show the internal OTU label, the genera label, log fold change between preterm and term models (where positive values show a higher change in preterm), the adjusted p-value by FDR (see section “Materials and Methods”) for the model and whether the taxa were “Significant” or “Reported” in the original cohorts. “Significant” represents a reported significant difference between term and PTB abundance. “Reported” represents abundance measurement reported in the original cohorts in the original papers.*

## Discussion

We carried out a comprehensive longitudinal meta-analysis of vaginal microbiome in PTB, merging five independent studies totaling 3,201 samples from 415 pregnant women that delivered at term or prematurely. Overall, looking at the t-SNE plot of normalized OTUs, partial clustering of the phenotypic groups was observed on a global scale. We found that the microbial within-sample variance in patients who deliver preterm is significantly different from those who deliver at term, with a higher microbial within-sample variance in the group that went on to deliver preterm. We looked at within-sample variance using a measure of sample variance. In general, variance is a method used in the gene expression field. This measure is also used by new emerging tools for microbiome analysis (Bradley and Pollard, 2017). The observed difference in within-sample variance agrees with two of the original studies (Hyman et al., 2014; Stout et al., 2017), whereas the others do not report differences in variance between patients who go on deliver preterm in comparison to those who deliver at term. DiGiulio et al. (2015) for example did not find changes in the variance throughout the pregnancy, but did find change between the pregnancy period to the post-partum period. Differences in vaginal composition between black and white non-pregnant women has been shown in the past (Fettweis et al., 2014). In our analysis we show that the higher within-sample variance in women who delivered prematurely is found in both populations, showing that this finding holds in different ethnic and racial groups.

We were also able to identify bacterial genera that are significantly associated with birth timing across all trimesters using linear mixed effects regression. First, an OTU representing *Lactobacillus* is found to be more abundant in patients who deliver at term across all trimesters. Since this finding is only at a genera level, further experiments are required here to carry out species and strain level identification. This finding is in agreement with other studies showing that the absence of *Lactobacillus* species can be used as a predictor for PTB (Usui et al., 2002; Petricevic et al., 2014). Second, we found several bacterial genera that are associated with PTB. Two of those, *Olsenella* and *Clostridium sensu stricto*, were not reported in the original studies we used in this meta-analysis. *Olsenella* is shown to be involved in BV, but not in PTB, and *Clostridium sensu stricto* was not reported as associated with BV or PTB.

An interesting attempt to predict PTB in the first trimester vaginal microbiome data was performed in a publication by Donders et al. (2009). The authors were able to show that women with normal vaginal microbiome had a 75% lower risk for PTB and that the absence of *Lactobacilli*, rather than BV, was the strongest risk factor. Based on the associations that we observe, with *Lactobacillus* as a protective genus found to be significantly higher in term patients across pregnancy trimesters, we would suggest that the lack of *Lactobacillus*, in combination with other anaerobic bacteria could be used as a stronger predictor for PTB when being tested across pregnancy trimesters.

There are a few limitations in this study. The first one reflects differences in 16S rDNA amplification. DiGiulio et al. (2015) the amplification was targeted toward the V3–V5 and V4 region of the 16S rRNA gene, in Callahan (Callahan et al., 2017) the V4 region was amplified, in Romero et al. (2014b), and Hyman et al. (2014) the V1–V3 region was amplified, and in Stout et al. (2017) both V1–V3 and V3–V5 regions were amplified. This may lead to unevenness in amplification of certain bacteria. One example of such known mismatch is amplification of *Gardnerella* (Hyman et al., 2005) and in both Romero et al. (2014b) and DiGiulio et al. (2015) the authors added primers to correct for this known bias. In our meta-analysis, we address this issue in several ways. The first is processing the data with a closed reference alignment method to address the amplification by different primers. In a closed-reference OTU picking process, reads are clustered against a reference sequence collection and any reads which do not hit a sequence in the reference sequence collection are excluded from downstream analyses and is the approach of choice if non-overlapping amplicons are compared. This way we limit the results only to known bacteria that is found in RDP, and the chance for mismatches that may arise from open-reference or *de novo* OTU picking decrease. Also, using a trustworthy reference database with full or almost full coverage to many of the 16S rRNA molecules, we decrease the chance of not identifying the same molecule by 2 different amplified regions from different studies. In addition, we carry out data normalization using ComBat to account for potential batch effects (Johnson et al., 2007). We show the removal of those batch effects by using gPCA and t-SNE plots. We apply linear mixed effects regression that takes into account patient-specific and cohort-specific effects (see section “Materials and Methods”) when carrying out association analysis. Finally, we have merged the OTUs into taxa to reduce variability and repeated the within-sample variance analysis. Those results show that genus level trends are in agreement with species level trends although the V region targeted is different for the five original cohorts.

Another potential limitation is the use of within-sample variance as the metric of difference between women who delivered at term vs. preterm. We are aware that this metric is not commonly employed microbiome studies, though it has been useful in gene expression analysis. Due to the nature of the OTU data we sought to model and the complexity of the normalization done by ComBat, we could not use any of the traditional metrics of alpha diversity. It is not a measure of richness, although it does quantify a particular notion of evenness because it is the sum of deviations of normalized OTU abundances from the mean abundance of a given sample. Furthermore, within-sample variance has a mathematical relationship to beta-diversity and provides a potentially useful measure of how unique a sample is in the data set. This within-sample variance approach produced results that are in agreement with the results in the individual studies we analyzed.

A last possible limitation is patient selection criteria. Patients in the combined studies come from different backgrounds in terms of geography, diet, lifestyle and other characteristics such as height, weight, medical conditions that may affect the results.

We included all available metadata we had in our models, but couldn’t account for missing information in the five studies we combined. While it is clear that other important variables such as prior medical conditions, prior pregnancies information, maternal age, periodontal disease, low maternal body-mass index, low socioeconomic and educational status and single marital status (Goldenberg et al., 2008) and maternal stress (Copper et al., 1996) have a huge impact on PTB, in most cohorts we used those variables do not exist. Also, we lack information about the mode of delivery (vaginal or c-section), about infections and other conditions during the pregnancy and have only partial information on medications given during pregnancy in a few of the studies. Having access to detailed phenotypic data can improve the models and allow us to explore other relevant questions in association with the microbial genera. Finally, the clinical definition of PTB may be different depending on the exact obstetrical definition that was used in the individual studies, however, the majority of the cohorts we investigated focused on spontaneous PTB and the signals that are robust despite the potential patient heterogeneity are more likely to be real as we have seen from prior gene expression meta-analysis studies (Dudley et al., 2009; Haynes et al., 2017; Sweeney et al., 2017). As more data becomes publicly available, we hope that additional standardization and meta-data availability will help address some of the aforementioned issues.

## Conclusion

We have shown that meta-analysis of the vaginal microbiome can shed light on outcomes of pregnancy, such as PTB. We first showed that there is a significant difference between the microbial within-sample variance in women who delivered prematurely when compared to patients who deliver at term. We then showed that this differential within-sample variance between women who deliver preterm in comparison to those who deliver at term is observed across pregnancy trimesters and is consistent across different racial or ethnic backgrounds. We also found that while *Lactobacillus* abundance is associated with term delivery, several genera are associated with PTB. Among those, two associations are novel. This result can inform future diagnostics and help in monitoring pregnancies with a simple swab done in the first trimester of pregnancy, and may in the future decrease the rates of PTB around the world.

## Data Availability Statement

Publicly available datasets were analyzed in this study. This data can be found here: SDY465 https://www.immport.org/shared/study/SDY465, PRJNA242473; https://www.ncbi.nlm.nih.gov/bioproject/PRJNA242473, PRJNA294119; https://www.ncbi.nlm.nih.gov/bioproject/PRJNA294119, PRJNA393472; https://www.ncbi.nlm.nih.gov/bioproject/PRJNA393472. The processed data was uploaded to ImmPort under study SDY1162 and to figshare (Kosti et al., 2019), together with relevant metadata.

## Ethics Statement

This study used publicly available data. The studies involving human participants were reviewed and approved at the time of original publications (please see references and data availability statement). Written informed consent was not necessary to obtain for this study.

## Author Contributions

IK, MS, and AB conceived and designed the analysis. IK refined and normalized the data, created the pipeline, and performed the data analysis and modeling. SL and KP designed the data modeling and association analysis. SL performed data modeling and association analysis. All authors contributed to writing and editing the manuscript.

## Conflict of Interest

The authors declare that the research was conducted in the absence of any commercial or financial relationships that could be construed as a potential conflict of interest.

## Footnotes

1 http://identifiers.org/ncbi/insdc.sra:SRP040750

2 http://identifiers.org/ncbi/insdc.sra:SRP115697

3 http://identifiers.org/ncbi/insdc.sra:SRP068239
