# Supplementary material for: Meta-Analysis of Vaginal Microbiome Data Provides New Insights Into Preterm Birth
Source: Front Microbiol. 2020 Apr 8;11:476. doi: 10.3389/fmicb.2020.00476 (PMC7156768; doi:10.3389/fmicb.2020.00476)
Supplement: Supplementary file 1 [file Presentation_1.PPTX]

## Slide 1
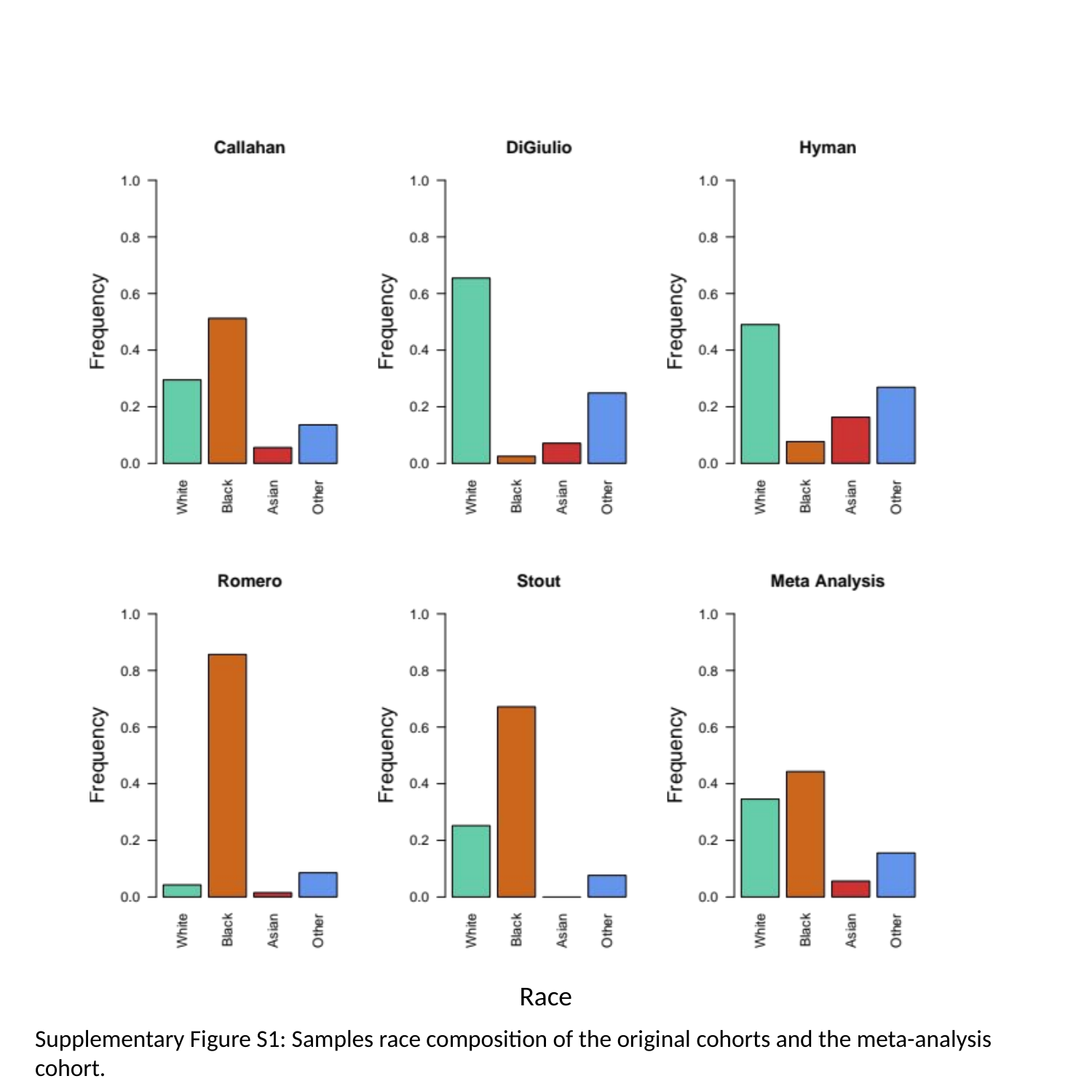

Race
Supplementary Figure S1: Samples race composition of the original cohorts and the meta-analysis cohort.

## Slide 2
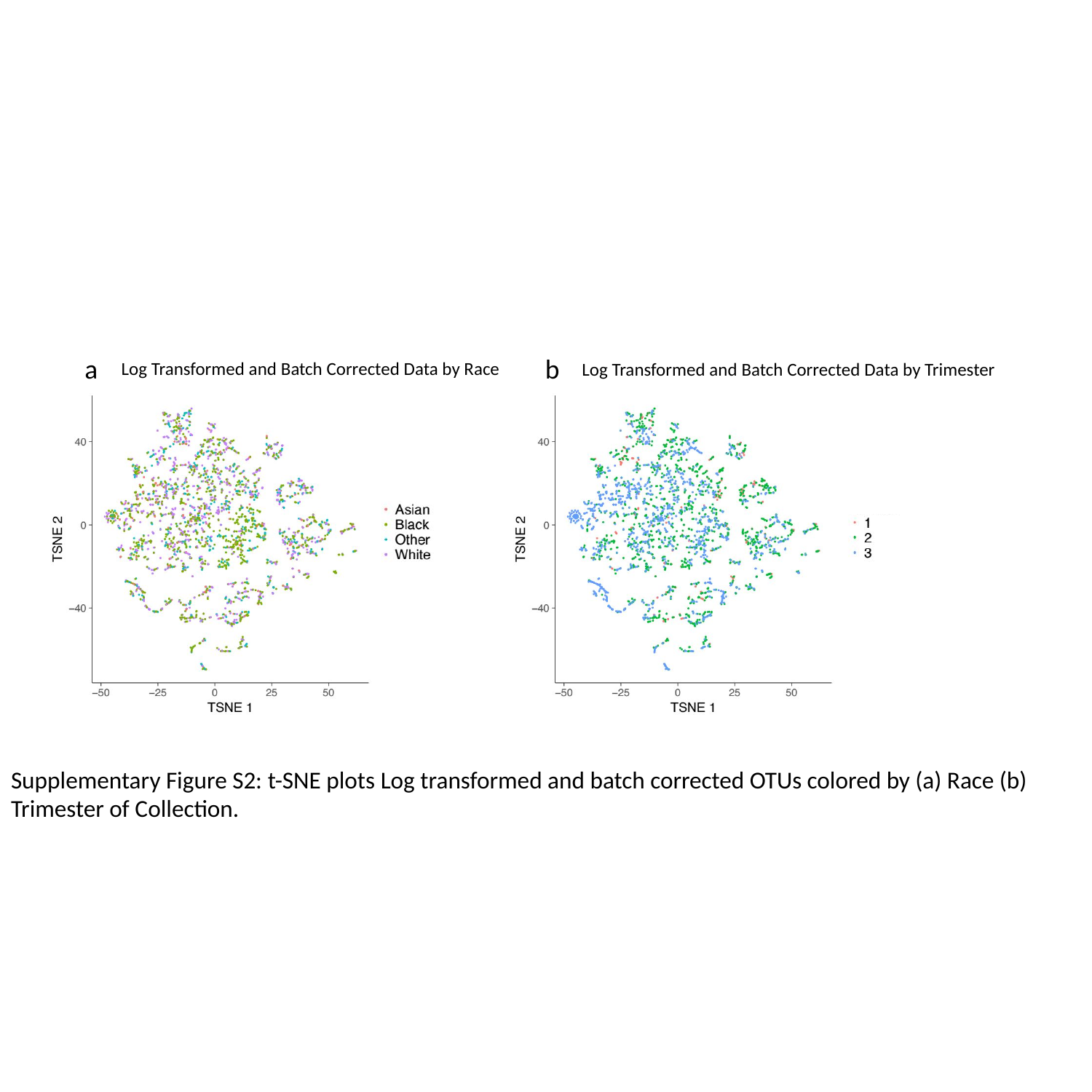

a
b
Log Transformed and Batch Corrected Data by Race
Log Transformed and Batch Corrected Data by Trimester
Supplementary Figure S2: t-SNE plots Log transformed and batch corrected OTUs colored by (a) Race (b) Trimester of Collection.

## Slide 3
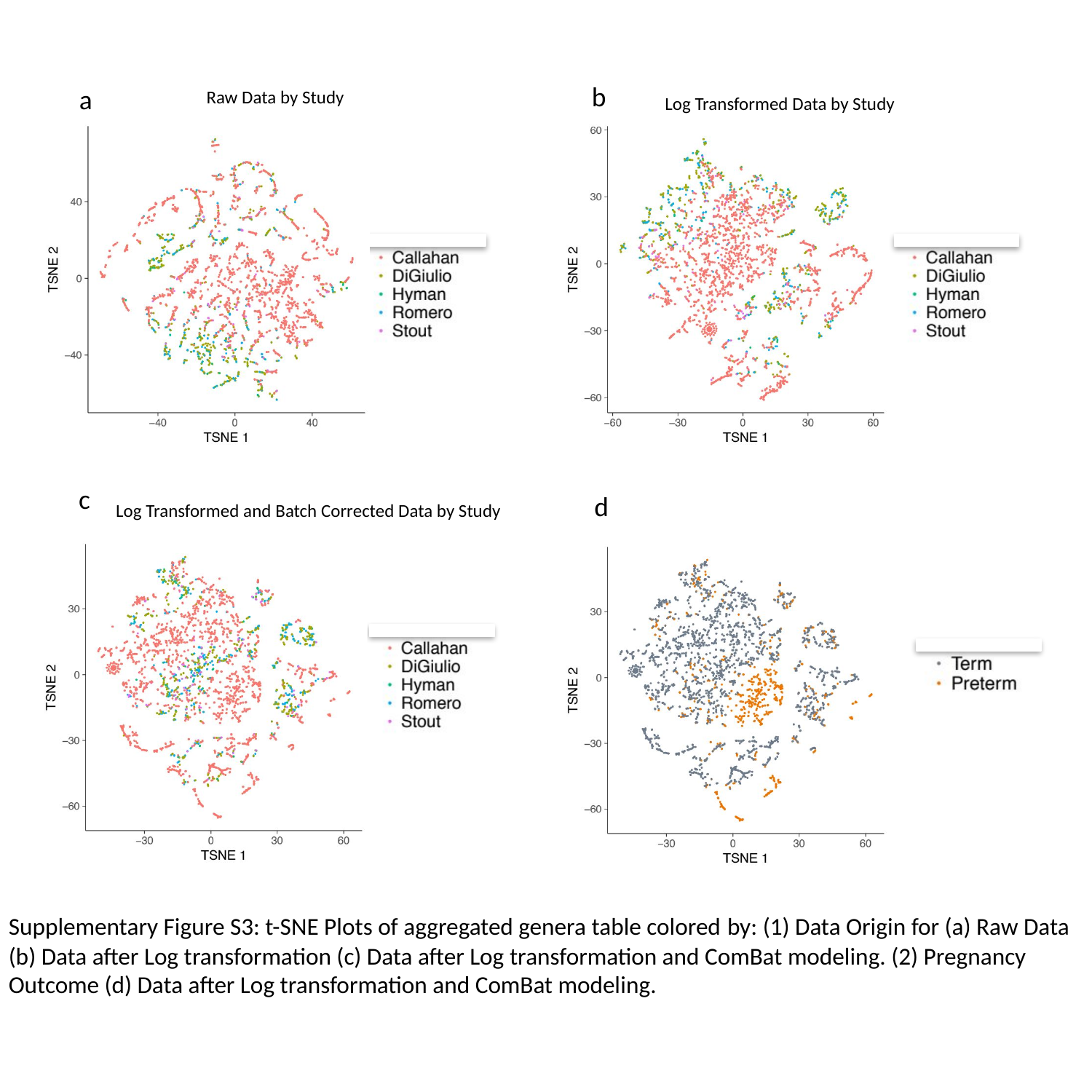

b
a
Raw Data by Study
Log Transformed Data by Study
c
d
Log Transformed and Batch Corrected Data by Study
Supplementary Figure S3: t-SNE Plots of aggregated genera table colored by: (1) Data Origin for (a) Raw Data (b) Data after Log transformation (c) Data after Log transformation and ComBat modeling. (2) Pregnancy Outcome (d) Data after Log transformation and ComBat modeling.

## Slide 4
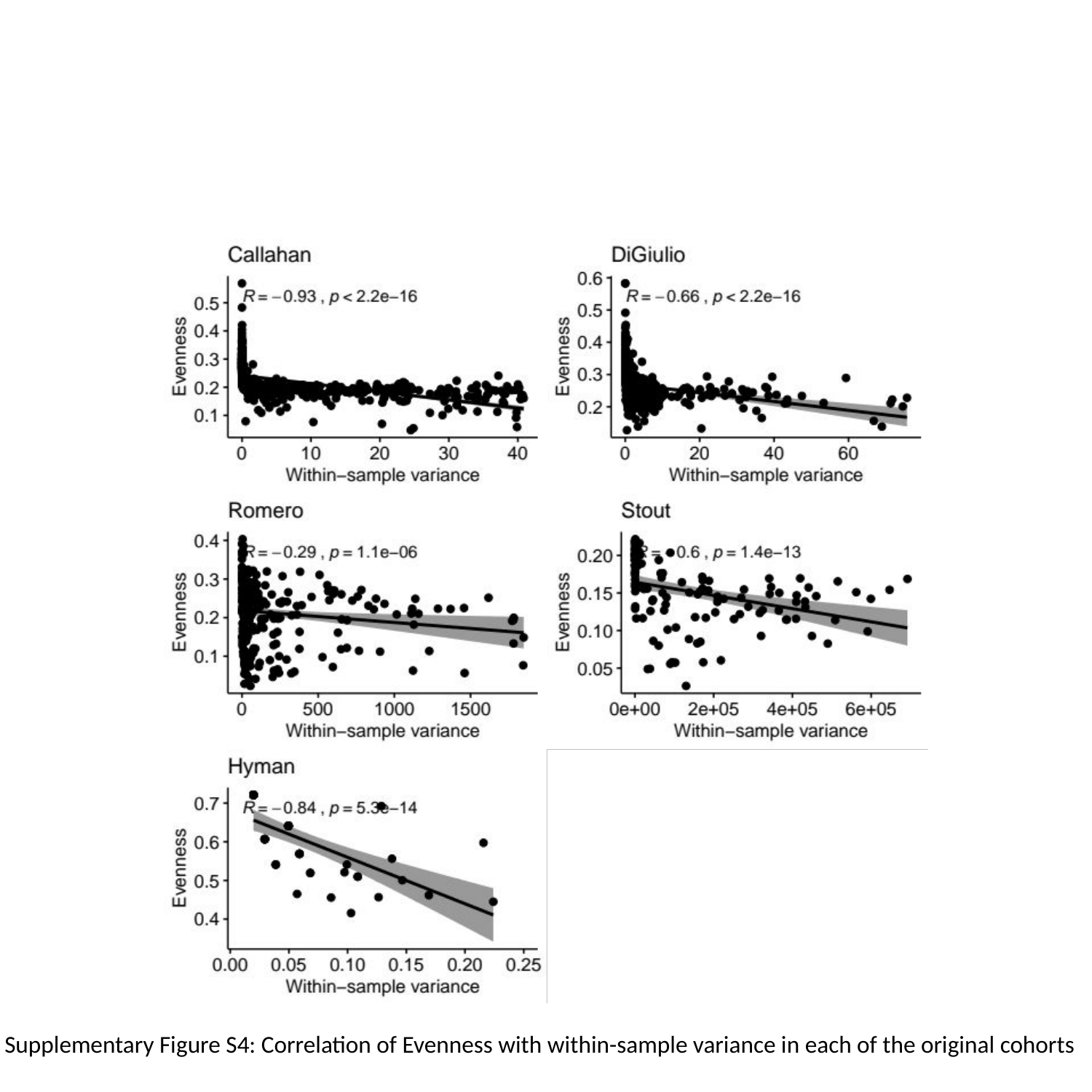

Supplementary Figure S4: Correlation of Evenness with within-sample variance in each of the original cohorts

## Slide 5
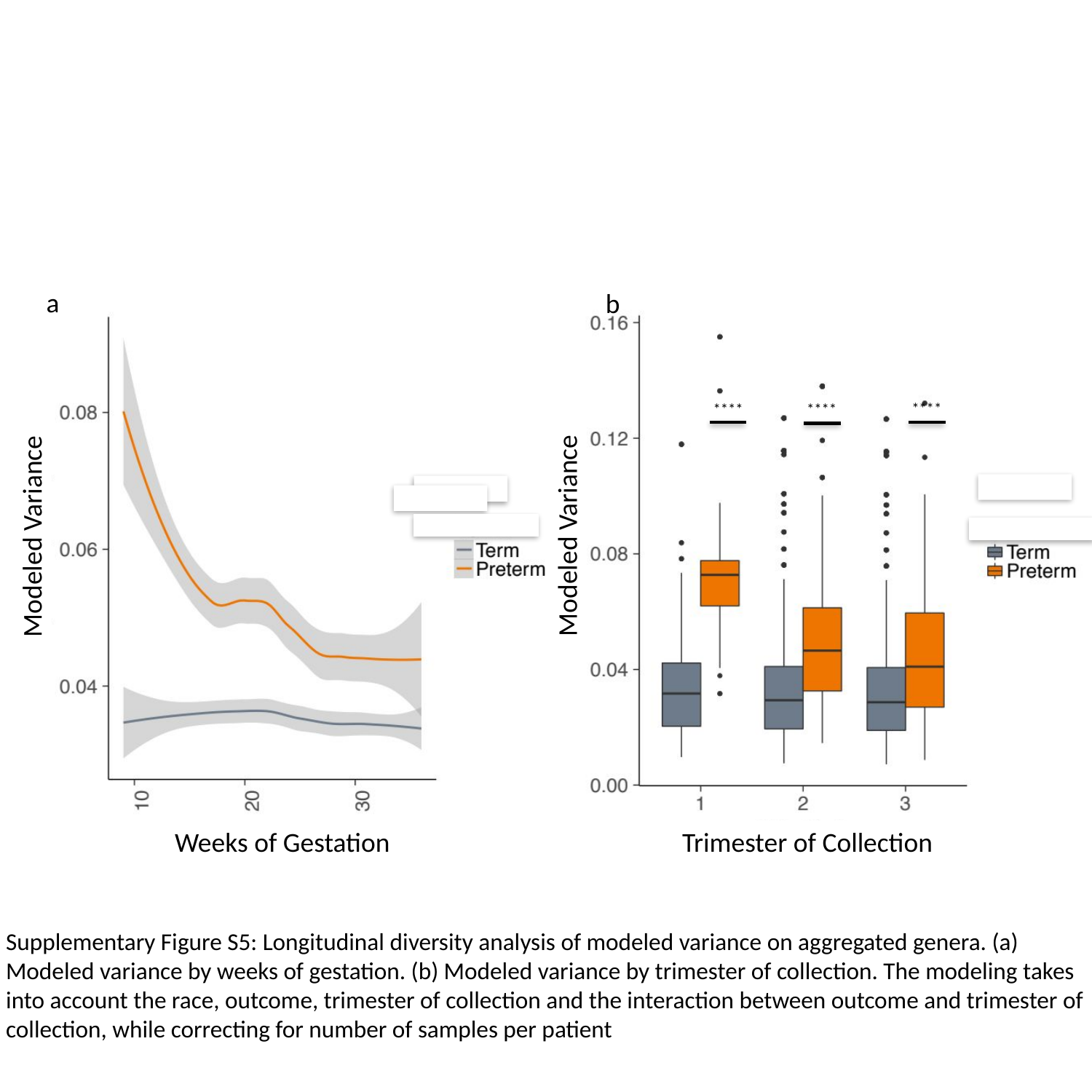

a
b
****
****
****
Modeled Variance
Modeled Variance
Trimester of Collection
Weeks of Gestation
Supplementary Figure S5: Longitudinal diversity analysis of modeled variance on aggregated genera. (a) Modeled variance by weeks of gestation. (b) Modeled variance by trimester of collection. The modeling takes into account the race, outcome, trimester of collection and the interaction between outcome and trimester of collection, while correcting for number of samples per patient

## Slide 6
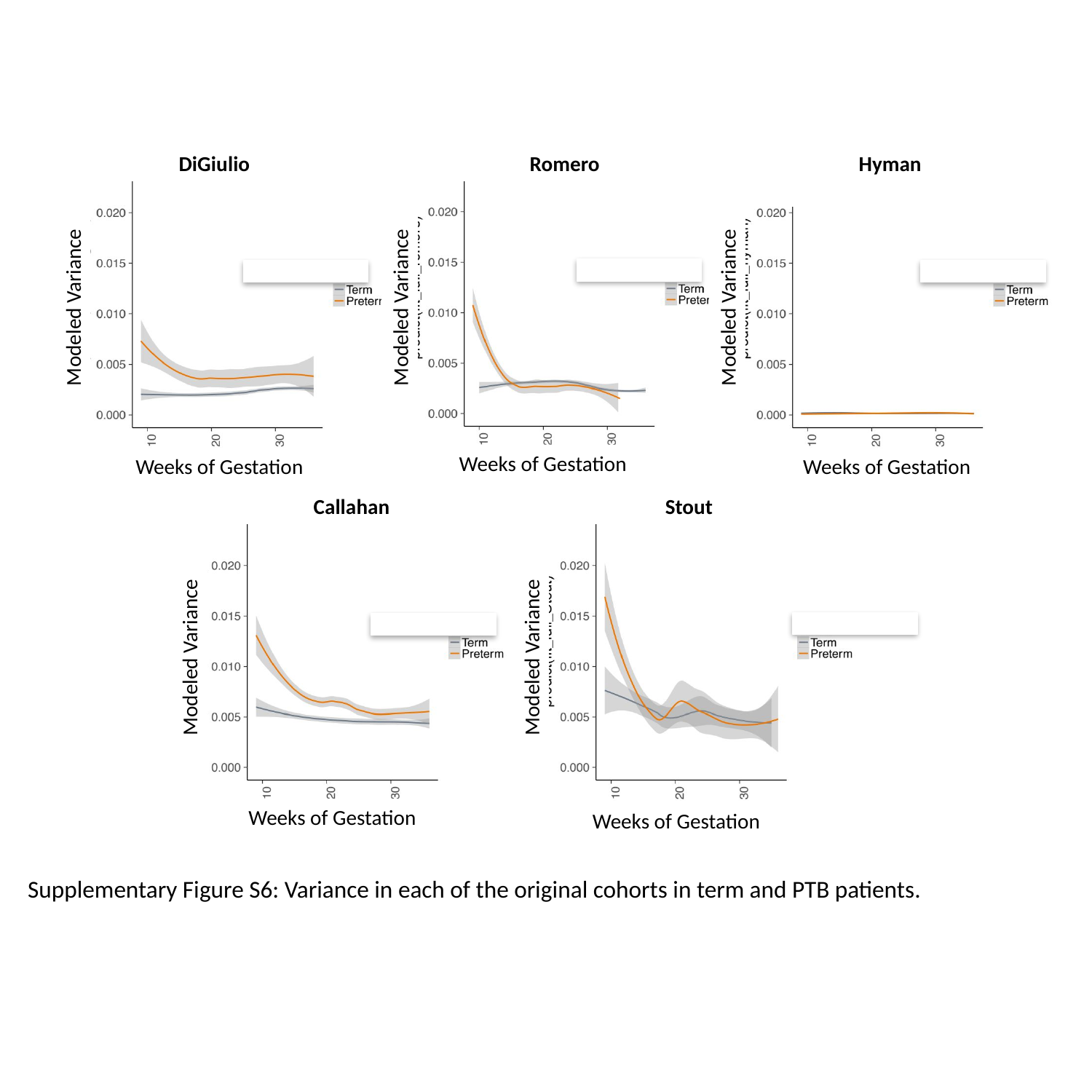

DiGiulio
Romero
Hyman
Modeled Variance
Modeled Variance
Modeled Variance
Weeks of Gestation
Weeks of Gestation
Weeks of Gestation
Callahan
Stout
Modeled Variance
Modeled Variance
Weeks of Gestation
Weeks of Gestation
Supplementary Figure S6: Variance in each of the original cohorts in term and PTB patients.

## Slide 7
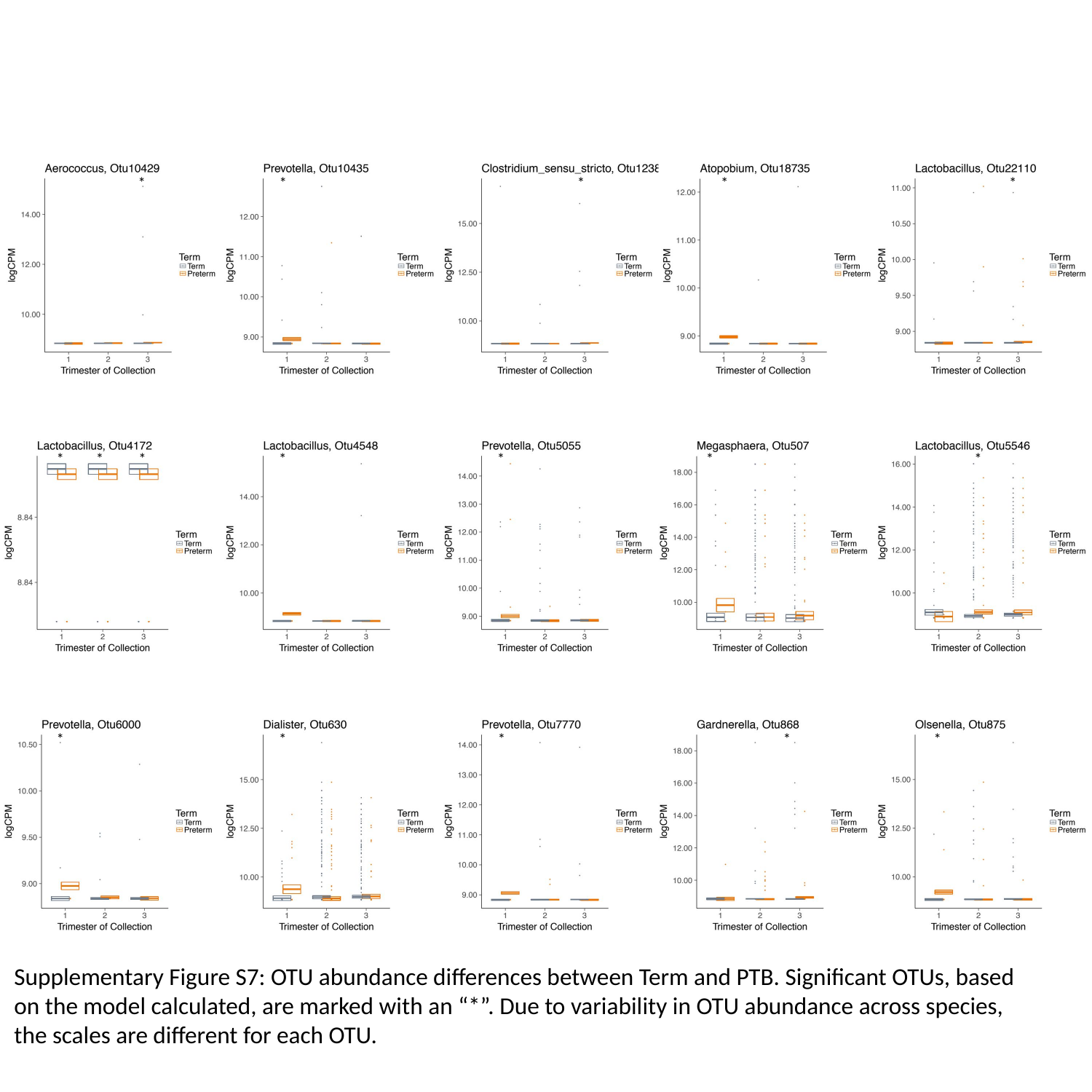

*
*
*
*
*
*
*
*
*
*
*
*
*
*
*
*
*
Supplementary Figure S7: OTU abundance differences between Term and PTB. Significant OTUs, based on the model calculated, are marked with an “*”. Due to variability in OTU abundance across species, the scales are different for each OTU.
